# Synthesis of Fluorescent Copper Nanomaterials and Detection of Bi^3+^

**DOI:** 10.3389/fchem.2022.899672

**Published:** 2022-05-25

**Authors:** Rihan Wu, Jun Ai, Lu Ga

**Affiliations:** ^1^ Inner Mongolia Key Laboratory of Environmental Chemistry, College of Chemistry and Enviromental Science, Inner Mongolia Normal University, Hohhot, China; ^2^ College of Pharmacy, Inner Mongolia Medical University, Hohhot, China

**Keywords:** Bi^3+^, detection, copper nanomaterials, fluorescence, glutathione

## Abstract

Based on the aggregation-induced luminescence of glutathione-protected non-noble metal copper nanoparticles (GSH-CuNPs), a fluorescence method for the rapid detection of bismuth (Bi^3 +^) was developed. The fluorescence intensity of GSH-CuNP solution is good, and the fluorescence can be quenched in the presence of Bi^3 +^. Based on this principle, a fluorescence mean for the admeasurement of Bi^3+^was built. The linear range was 0–100 mmol/L, and the detection limit was 10 mmol/L. The method is simple, rapid, and selective and can be used for the qualitative detection of Bi^3 +^.

## Introduction

Due to the serious impact of metal ions on the environment and human health, the detection of metal ions has been a hot research topic. (Brower et al., 1997; Aragay et al., 2011). These species come from anthropological and natural resources and are found in soil, reservoir water, and marine environments (Yoshizaki and Tomida, 2000; Gans et al., 2005; Ghaedi et al., 2009). They accumulate in plants, meat, and other foods (Gill, 1983; Oyaro et al., 2007; Sud et al., 2008). Through these different absorption channels, these ions can gather in the developing brain, destroy the secondary structure of proteins/peptides, and lead to debilitating diseases (Stohs and Bagchi, 1995; Järup, 2003; Sharma and Dietz, 2009). Specifically, the local accumulation of Zn^2+^ in the brain leads to the rapid induction of the amyloid structure in Alzheimer’s disease (Bush et al., 1994; Lee et al., 2004). Due to the obstinacy of heavy metal ions in the environment and the resistance to conventional filtration methods, heavy metal ion pollution has plagued most developed countries (Fu and Wang, 2011). The harmful effects of heavy metal ions have prompted extensive research on their sensitive detection (Alexander et al., 2000; Ferreira et al., 2004; Korolczuk et al., 2005; Lee et al., 2008; Li et al., 2012). Acute bismuth poisoning is mainly due to oral entry; the patient will have nausea, vomiting, salivation, tongue and throat pain, abdominal pain, diarrhea, black stool with blood, skin and mucous membrane bleeding, headache, spasm, etc. Liver and kidney damage can lead to jaundice, urine protein and tube type, and even acute liver and kidney failure. For those allergic to bismuth salt, fever, rash, acute hemolysis, and exfoliative dermatitis may occur after intramuscular injection. Long-term application of bismuth can cause multiple neuritis, stomatitis, gingival swelling, pigmentation of the oral mucosa, and a black line on the gingiva. The X-ray film of the long bone end of the patient showed a white band, which was similar to that of lead poisoning cases.

As the next generation of materials, nanomaterials have shown great application prospects in many fields, such as catalysis, photonics, environmental detection, biomedical diagnosis, and treatment (Nie et al., 2010; Chen et al., 2012; Lu and Yin, 2012; Schroeder et al., 2012). This prospect comes from the unique properties of these nanomaterials, such as their plasma and quantum confinement effects, which are largely dependent on morphology (such as shape and surface structure) (Xia et al., 2009). Fluorescent metal nanoclusters (Au, Ag, and CuNCs) have attracted much attention due to their ultra-small size, quantum confinement, high photostability, and biocompatibility (Zheng et al., 2004; Burda et al., 2005; Zhu et al., 2008; Xie et al., 2009; Bain et al., 2015; Barman et al., 2016; Jin et al., 2016; Bain et al., 2017; Chakraborty and Pradeep, 2017; Kundu and Patra, 2017; Cao et al. 2014). Water soluble CuNCs were prepared from tannic acid, and the fluorescence quenching induced by the electron transfer mechanism was used to realize the high sensitivity detection of Fe^3 +^. At present, the detection of metal ions by fluorescent nanoclusters is mostly based on the fluorescence quenching mechanism. Since there are many substances that inhibit fluorescence, fluorescence quenching is a common phenomenon and has a great influence on selectivity or sensitivity (Chen et al., 2016). Fluorescence AgNCs were synthesized with glutathione (GSH) as a protective agent, and S^2−^ quenched the fluorescence of silver nanomaterials to detect S^2−^. In the work by Feng et al. (2015), branched polyethyleneimine (bpei)-terminated CuNCs were synthesized by using bpei as the template and ascorbic acid as the reducing agent. The results display that the luminous intensity of CuNCs reaches its maximum value at 360 and 430 nm. Even in a high-salt medium, it has great water solubility, high stability, and photostability. The results show that Fe^3+^can significantly quench the fluorescence of bpei CuNCs by electron transfer, while other common anions and cations have little effect on the luminous intensity of CuNCs. Based on these, an ultrasensitive and super selective Fe^3+^fluorescence sensor was built and applied for measure of Fe^3+^ in human urine and water.

In this experiment, Cu nanoparticles were prepared by an easy template method. GSH is used as the protective agent and template of CuNPs, and the synthesized high fluorescence CuNPs can detect bismuth ions. When bismuth ions were added into the system, the fluorescence of Cu nanoparticles was inhibited. Moreover, among the common inorganic metal cations and anions, only Bi^3+^ can trigger the significant fluorescence quenching effect of copper nanomaterials. The nanomaterial sensor has good linear range, stability, and high selectivity and has great potential in the field of metal ion sensing and detection.

## Materials and Methods

### Materials

Glutathione (GSH), CuSO_4_, Cu (NO_3_)_2_ 3H_2_O, ZnSO_4_·7H_2_O (Analytically Pure, Tianjin FengChuan Chemical Reagent Technology Co., Ltd.), FeCl_2_·4H_2_O (Analytical Pure, Beijing Shangle Chemical Plant), Bi(NO_3_)_3_·3H_2_O, NaCl, KCl (Analytical Pure, Tianjin Beilian Fine Chemicals Development Co., Ltd.), Ni (NO_3_)_2_·6H_2_O (Analytical Pure, Beijing 5671 Chemical Plant), NaOH (Analytically Pure, Tianjin Shengao Chemical Reagent Co., Ltd.), MnSO_4_·H_2_O (Analytical Reagent, No.4 Chemical Plant, Chaoyang District, Beijing), Cd(NO_3_)_2_ (Analytical Pure, Beijing Chemical Plant), NH_4_Cl (Analytical Pure, Tianjin People’s Chemical Plant), Na_2_SiO_3_·9H_2_O (Analytical Pure, Chemical Plant of Beijing Yizhuang Middle School), NaNO_2_ (Analytical Reagent, Tianjin Public Private Joint Venture Chemical Reagent Factory No.1), KI (Analytical Reagent, Baoding Chemical Reagent Factory, Hebei Province), Na_2_SiO_3_·9H_2_O (Analytical Pure, Chemical Plant of Beijing Yizhuang Middle School), CoCl_2_·6H_2_O (Analysys, Shanghai Public Private Joint Venture China Trade Factory), NaF (Analytical Pure, Beijing Public and Private Chemical Plant), and ultrapure water (about 18.25 m Ω).

### Instruments

JEOL-2100f high resolution transmission electron microscope (equipped with xflash, Bruker, Germany), 5030t X-ray spectrometer (Japan Electronics Co., Ltd.), F-4600 fluorescence spectrophotometer (Hitachi high tech company), PHS-3c pH meter (Shanghai Yidian Scientific Instrument Co., Ltd.), d1008 series handheld centrifuge (Beijing Dalong Xingchuang Experimental Instrument Co., Ltd.), zf-7a portable UV detection lamp (Shanghai Guanghao analyzer Co., Ltd.), manual (adjustable and fixed) pipette (Beijing Dalong Xingchuang Experimental Instrument Co., Ltd.), and HJ-4A constant temperature magnetic heating agitator (Jiangsu Jintan Honghua instrument factory).

### Synthesis of Copper Nanomaterials

Cu nanoparticles were prepared by blending 0.5 ml 50 × 10−^3^ M Cu (NO_3_) _2_ ·3H_2_O with 2.5 ml of 50 × 10−^3^ M glutathione (GSH) and 2.0 ml of water, adjusting to pH 2.7 with 2 M NaOH, and centrifuging for 20 min to obtain GSH-CuNPs. Finally, it was stored at 4°C for subsequent experiments.

### Detection of a Trivalent Bismuth Ion

The prepared GSH-CuNPs were put into a 1.5 ml centrifuge tube at 450 μ L, and then 50 μ L Bi^3+^ ion solutions with different levels were added. The reaction time was 15 min at room temperature. The fluorescence intensity at 471 nm was measured.

## Results and Discussion

### Synthesis Principle of GSH-CuNPs

**FIGURE 1:**
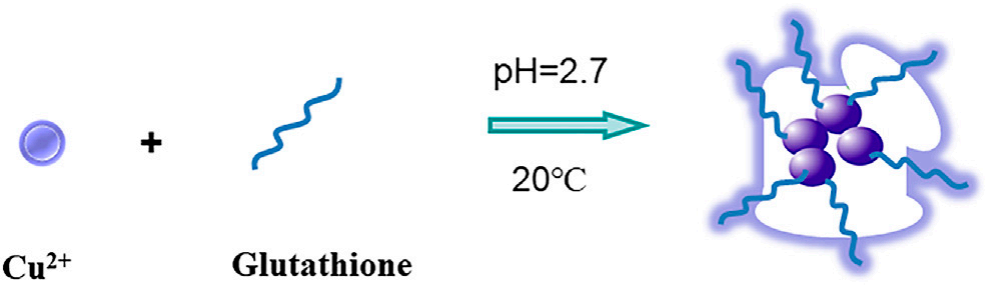
Sketch map of the GSH-CuNPs.

In this test, the synthesis method of GSH-CuNPs is shown in Figure 1. GSH-CuNPs with hyperfluorescence were prepared at room temperature with glutathione as the protective agent and reducing agent. When Bi^3+^ was added into the GSH-CuNP system, Bi^3+^ would destroy GSH-CuNPs and cause aggregation of GSH-CuNPs, resulting in fluorescence fading. Therefore, based on the trivalent bismuth ions, we can effectively inhibit the fluorescence of GSH-CuNPs so as to realize the detection of bismuth ions.

### Characterization of Copper Nanomaterials

**FIGURE 2:**
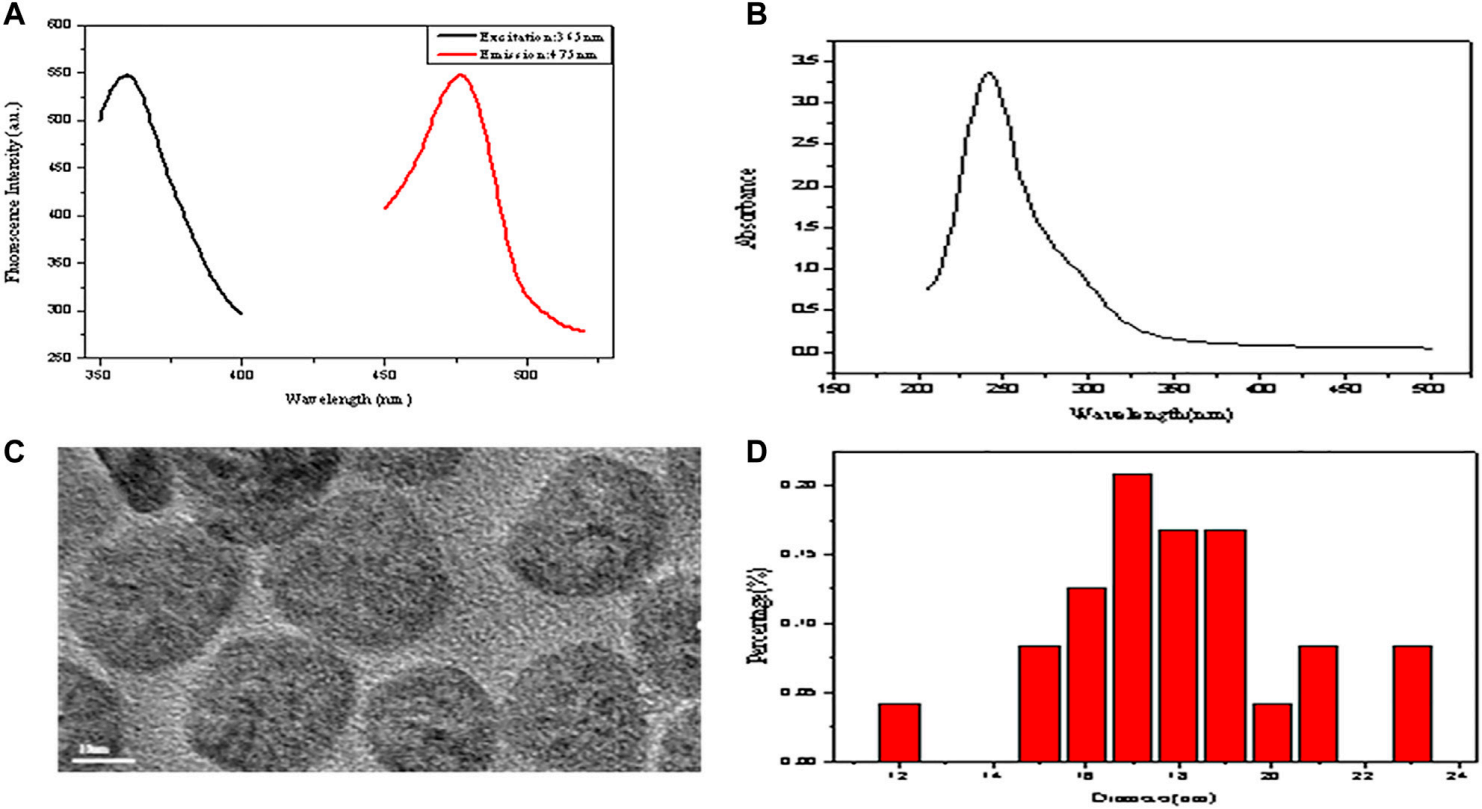
**(A)** Fluorescence spectra of GSH-CuNPs; **(B)** UV–vis spectra of GSH-CuNPs; **(C)** TEM image of GSH-CuNPs; **(D)** size-distribution histogram of GSH-CuNPs.

The fluorescence spectra of GSH-CuNPs are shown in Figure 2A. The emission and excitation of GSH-CuNPs are at 475 and 365 nm, respectively. As shown in Figure 2B, Cu nanoparticles have a small UV absorption spectrum, which also proves the successful synthesis of copper nanomaterials. Figure 2C exhibits the TEM image of GSH-CuNPs with small particle size and uniform distribution. Figure 2D exhibits the particle size spreading of GSH-CuNPs. The average diameter of GSH-CuNPs is about 17.0 ± 0.2 nm. These findings suggest that the synthesized GSH-CuNPs have the characteristics of great fluorescence, equality, dispersibility, and granule dimensions, indicating the successful synthesis of hyperfluorescence GSH-CuNPs.

### Stability of Copper Nanomaterials

**FIGURE 3:**
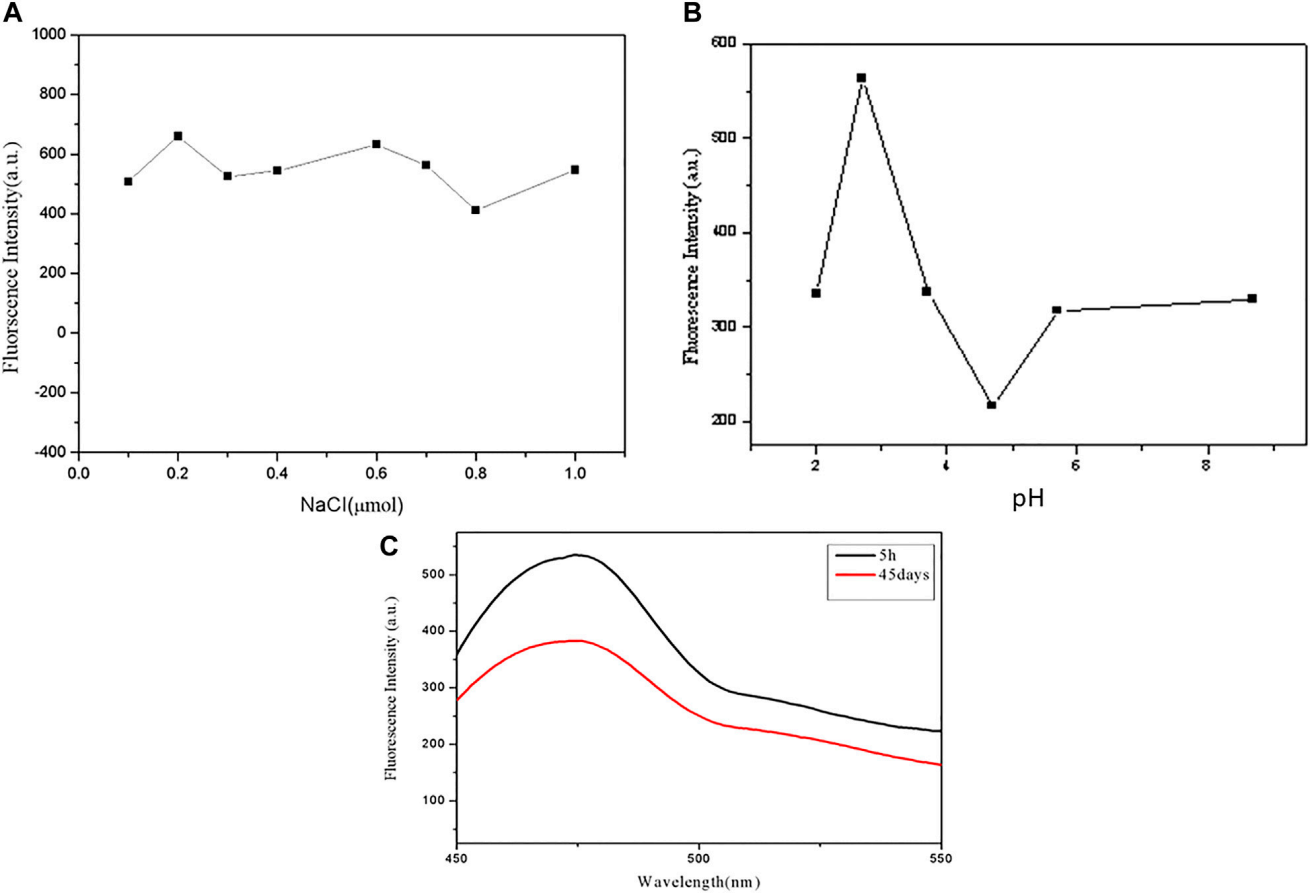
**(A)** Fluorescence magnitude of GSH-CuNPs; **(B)** effect of pH value on GSH-CuNP fluorescence intensity; **(C)** GSH-CuNPs can be stored for 1 month at 4°C.

We study the stability of GSH-CuNPs under different conditions. As revealed in Figure 3A, the fluorescence magnitude of GSH-CuNPs remains unchanged when a sodium chloride solution of different concentrations is added. As exhibited in Figure 3B, when the pH value of CuNPs was adjusted, it was found that the fluorescence magnitude of CuNPs was affected by pH. When pH is 2.7, GSH-CuNPs have a higher fluorescence magnitude. As shown in Figure 3C, the fluorescence intensity of GSH-CuNPs remains unchanged with time. As we can see, compound GSH-CuNPs can be stored for about 45 days at 4°C. The aforementioned data prove that GSH-CuNPs have good storage stability and good optical stability.

### Selectivity and Sensing Properties of Copper Nanomaterials

**FIGURE 4:**
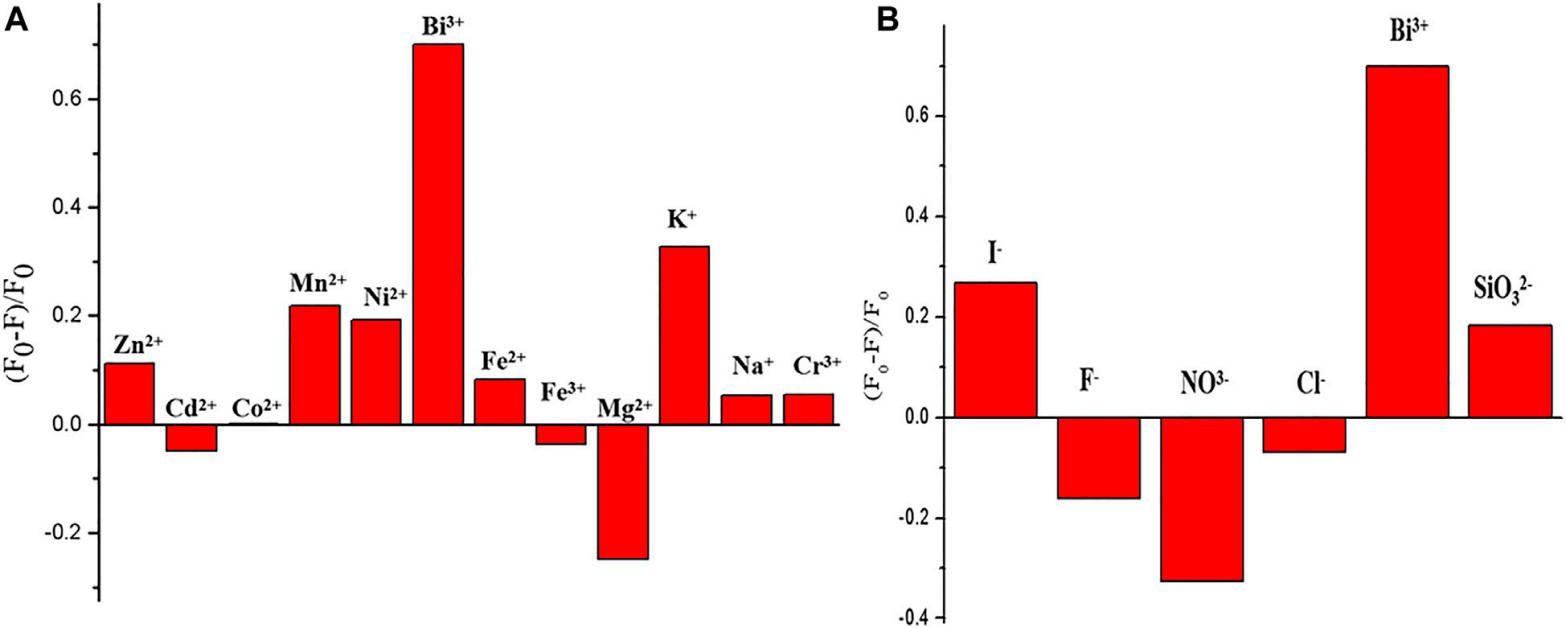
Fluorescence intensity of GSH-CuNPs at 475 nm under the existence of various cations **(A)** and anions **(B)**.

A series of different cations (such as CO^2 +^, Mg^2+^, Cd^2 +^, K^+^, Na^+^, Bi^3+^, and Fe^3+^) and anions (such as I^-^ and F^-^) with identical concentration (100 mmol L^−1^) were tested. As shown in Figures 4A, B, Bi^3+^ can quench GSH-CuNPs, and the other ions have little effect on GSH-CuNPs. The results show that the technique has higher selectivity for the gauging of Bi^3+^.

### Analysis of Sensing Properties of Copper Nanomaterials

**FIGURE 5:**
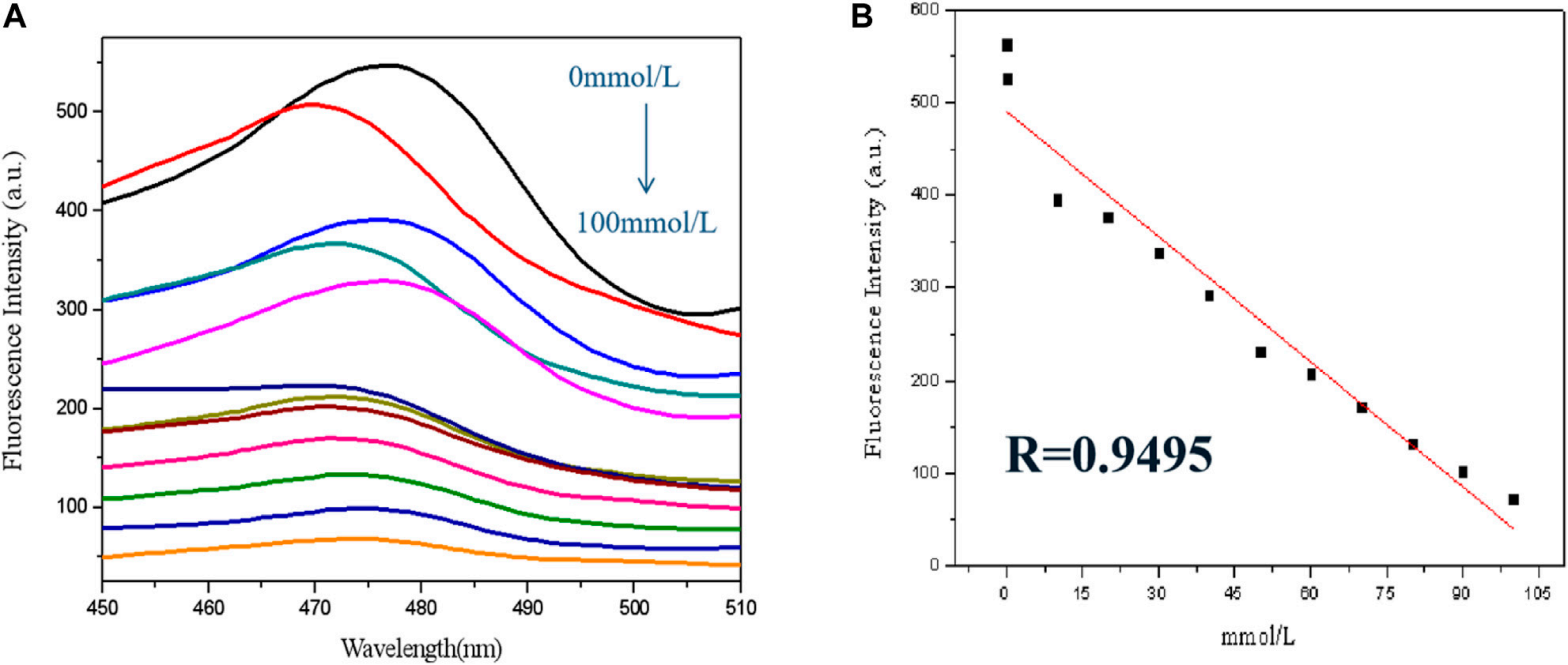
**(A)** GSH-CuNP fluorescence spectra with different concentration of bismuth ions; **(B)** linear relationship between fluorescence intensity of GSH-CuNPs and concentration of Bi^3+^.

In this trial run, a few bismuth ions with different levels were used for sensor analysis. As revealed in Figure 5A, the fluorescence of GSH-CuNPs was found by inch inhibition with the increase of bismuth ion concentration (0 mmol l^−1^–100 μ mol L^−1^). As revealed in Figure 5B, Bi^3+^ concentration has a good linear relationship in the range of 0 μ mol l^−1^–100 μ mol L^−1^ (R = 0.9495), and the detection limit is 10 mmol L^−1^.

## Conclusion

In view of the fluorescence inhibition mechanization, a fluorescent analysis means for the detection of Bi^3+^ was built by using GSH-CuNPs as the fluorescence probe and Bi^3+^ as the quenching agent. With the increase of Bi^3+^ concentration, the fluorescence of Cu nanoparticles is inhibited in diverse degrees. The results showed that the fluorescence quenching linear relationship was good in the range of 0 μ mol l^−1^–100 μ mol L^−1^ (R = 0.9495). The GSH-CuNPs can be used for selective detection of bismuth ions.

## Data Availability

The original contributions presented in the study are included in the article/Supplementary Material, further inquiries can be directed to the corresponding authors.
